# Physiological Characteristics of Putative Enterobacteria Associated with Meat and Fish Available in Southern Brazilian Retail Markets: Antimicrobial Susceptibility, Toxic Metal Tolerance and Expression of Efflux Pumps

**DOI:** 10.3390/antibiotics11121677

**Published:** 2022-11-22

**Authors:** Renata da Costa Barros Silva, Jéssica Andrade, Vanessa Cordeiro Dias, Jéssica Carla Sequeto, Nayara Felga Santos, Vânia Lúcia da Silva, Cláudio Galuppo Diniz

**Affiliations:** Laboratory of Microbial Physiology and Molecular Genetics, Center for Studies in Microbiology, Department of Parasitology, Microbiology and Immunology, Federal University of Juiz de Fora, Juiz de Fora 36036-330, Brazil

**Keywords:** meat, antimicrobials, resistance, *Enterobacteriaceae*, toxic metals

## Abstract

Multidrug-resistant (MDR) mesophilic facultatively anaerobic Gram-negative rods are a public health issue and their spread from animal-source foods to humans is of concern worldwide. Hence, the aim of this study was to examine the antibiotic susceptibility patterns and physiological aspects of such rods, including their tolerance to toxic metals and the screening of efflux pumps expressing isolates among enterobacteria isolated from meat (chicken, beef and pork) and fish samples acquired from retail establishments in a Brazilian urban Centre of over 2,300,000 inhabitants. The study revealed that 62.9% of isolated bacteria were resistant to at least one antimicrobial, of which 32.3% and 8.1% were resistant to one and two of the tested drugs, respectively. A resistance of up to six antimicrobials was also observed (0.9%). Out of the total amount, 22.7% were classified as MDR. Chicken was the meat that harbored most MDR isolates, and fish harbored the least. It was not possible to distinguish the different types of meat or fish considering the resistance patterns. The MDR isolates showed a higher tolerance to mercury and cadmium salts and the increased activity of the efflux mechanisms compared to other susceptible or resistant strains. In One Health. the perspective occurrence of putative MDR bacteria in fresh meat and fish draws attention to the antimicrobial resistance phenomenon in an open environment.

## 1. Introduction

Animal-source foods (ASF) are an important component of the human diet and a significant source of high-quality nutrients, but their consumption is a key point of exposure to foodborne infections [1,2,3,4]. The Centers for Disease Control and Prevention (CDC) estimates that close to 48 million people in the USA are infected by foodborne pathogens annually, leading to 128,000 hospitalizations [5]. In addition to the social impact, the annual economic burdens are estimated to be of up to USD 90 billion and together, these facts highlight the importance of preemptive measures to improve food safety as a whole [6].

Among the etiological agents of foodborne illnesses, the enterobacteria species, formerly characterized as *Enterobacteriaceaeae* but now included in *Enterobacteriaceae*, *Morganellaceae* and *Yersiniaceae* families (order *Enterobacterales*), are some of the most frequent [3,7,8,9]. Species notably recognized as foodborne pathogens include *Escherichia coli*, *Yersinia enterocolitica*, *Salmonella* spp. And *Shigella* spp., among others. They are commonly associated with gastrointestinal distress and the infections are in general self-limiting. Nonetheless, these infections may evolve to a systemic disease and death might be an outcome, particularly in vulnerable groups such as the elderly, children, pregnant women and the immunocompromised [10]. Extraintestinal infections can also occur, including blood stream infections, meningitis, post-infectious reactive arthritis, spleen infection, chronic kidney disease, hemolytic uremic syndrome and osteomyelitis [8].

Against this background, infections caused by antimicrobial resistant (AMR) enterobacteria have significant clinical and economic impacts, and their presence in meat constitutes a major threat to public health. Several accounts of multidrug resistance (MDR) among this group in different types of meat have been recorded all over the world, indicating that the issue is generalized [1,2,11]. A resistance to antibiotics is a natural phenomenon, but the misuse and exaggerated application of these drugs in human health and animal husbandry have led to the spread of AMR bacteria even in the food chain [12]. Moreover, there is growing evidence of the co-selection of AMR in bacteria exposed to biocides and toxic metals used by the agri-food industry [13]. Once in the food matrix or human gastrointestinal tract, AMR bacteria could transfer genetic resistance determinants among themselves via genetic mobile elements, which aggravates even further the matter of food safety [14,15]. For this reason, it is of utmost importance to tackle the issue of antimicrobial resistance with a One Health approach, acknowledging the inextricable nature of human, animal and environmental health [16].

Controlling the spread of enterobacteria found in meats retailed domestically and internationally is essential for reducing the economic and social impact of the diseases caused by them and improving the food security for the population. Considering Brazil’s position as one of the largest meat exporters worldwide, vigilance studies are crucial due to the implications of the spread of *Enterobacterales* members on public health and the potential for the dissemination of MDR strains. This study aims to analyze the physiological aspects and antibiotic susceptibility patterns of representative enterobacteria isolated from chicken, beef, pork and fish available in a retail market as low-processed food of an animal origin.

## 2. Results

### 2.1. Bacterial Isolation and Total Viable Counts

Overall, 436 bacteria samples were isolated and presumptively characterized as enterobacteria. Gram-negative non-enterobacteria and Gram-positive bacteria were stored under −80 °C for further studies. Regarding the total bacteria count in the evaluated meat and fish samples, the mean viable counts (log10 CFU/g) were 5.92 BA, 4.97 in EMB and 4.75 in MSA. Detailed viable counts are presented in Table 1. The bacterial quantification showed no statistically significant differences between the means obtained from the total count of microorganisms, the Gram-positive cocci counts and Gram-negative rods counts derived from poultry, beef and pork samples. However, the same did not happen for the counts obtained from fish samples, where the mean of the Gram-positive cocci counts was significantly lower than that of the other samples (*p* < 0.05).

### 2.2. Antimicrobial Susceptibility Patterns

With an exception for gentamicin and meropenem, a resistance to all the tested antimicrobials was observed. Beta-lactam drugs were the least effective, with resistance rates of 39.1% against cefazolin, 30.5% against ampicillin, followed by resistance rates of 18.1% and 17.7% against amoxicillin/clavulanic acid and cefoxitin, respectively (Table 2). In lower rates, a resistance was also observed against tetracycline (6.3%), chloramphenicol (3.3%), sulphazotrim (3.0%), ciprofloxacin (1.6%), aztreonam (1.4%), levofloxacin (1.1%), ceftazidime (1.2%), ceftriaxone (0.5%), cefepime (0.5%) and amikacin (0.2%).

Although a resistance to cloramphenicol, sulphazotrim and tetracycline was observed for bacteria isolates from all types of meat and fish, it was more frequent in representative bacteria from pork meat (Table 2). The same is also true for ampicillin and amoxicillin/clavulanic acid in isolates derived from chicken. Amikacin was not effective in one isolate derived from beef, and aztreonam in some isolates derived from chicken and fish. A resistance to ceftriaxone and cefepime was observed only among bacteria isolated from fish samples, albeit their resistance rates for the other tested drugs were generally lower compared to the isolates from other meats (Table 2).

Out of all the 436 isolates, 271 (62.9%) were resistant to at least one of the antimicrobial drugs, 32.3% of which were resistant to only one and 8.1% to two. According to the definition of multidrug resistance proposed by Magiorakos et al. [17]. For the formerly known *Enterobacteriaceae* (2012), it was found that isolates resistant to three or more of the tested antimicrobials (22.7%) could be considered MDR and derived from high-risk sources of contamination. Among this group, 7.9% of the isolates were resistant to three drugs, 11.4% to four, 2.3% to five and 0.9% to six of the tested antimicrobials (Table 3). One isolate exhibited the resistance phenotype ATM-CAZ (0.2%) and was considered MDR by classical definition.

Isolates resistant to at least one of the tested antimicrobials represented 72.6% of the bacteria isolates from beef samples, 71.5% of the ones from chicken, 67.8% of the ones from pork and 48.4% of the ones from fish. Chicken was the meat from which fewer isolates resistant to one drug (26.4%) were obtained when compared to other meats and fish, however it was the one that harbored the most considered multi-resistant bacteria (34.3%). Unlike isolates from chicken, beef and pork that presented an MDR to up to six antimicrobials, a resistance to more than five drugs was not observed for the fish-isolated bacteria. Indeed, considering the fish-isolated bacteria, this microbial group comprised the least amount of MDR strains (11.7%) (Table 3).

**Figure 1:**
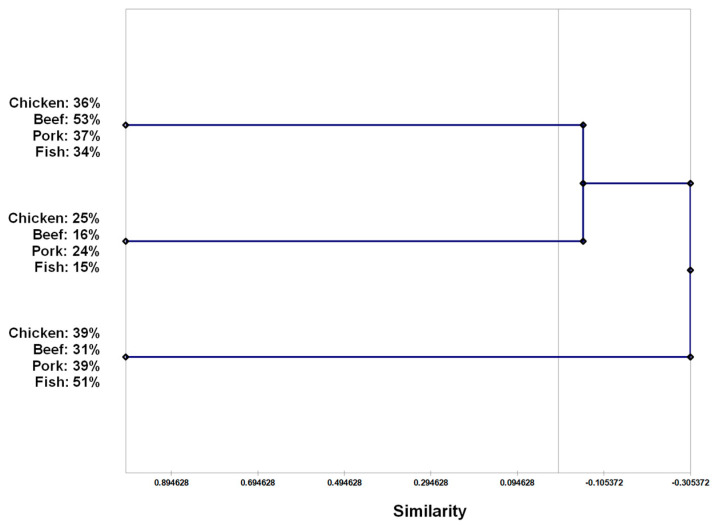
Similarity phenogram from hierarchical clustering analysis by UPGMA (unweighted pair group method with arithmetic mean) with bootstrap of 1000× based on antimicrobial resistance or antimicrobial susceptibility of mesophilic facultatively anaerobic Gram-negative rods isolated from meat and fish samples.

From the antimicrobial susceptibility patters, the hierarchical clustering analysis by UPGMA showed that the 436 bacteria isolates were divided into 3 distinct groups: A1, A2 and A3. Group A1 (*n* = 171) was comprised of 36% of isolates which derived from chicken samples, 53% from beef samples, 37% from pork samples and 34% from fish samples. A2 (*n* = 84) was a group with 25% of the isolates from chicken samples, 16% from beef, 24% from pork and 15% from fish. In group A3 (*n* = 181), 39% of isolates related to chicken samples, 31% related to beef samples, 39% related to pork samples and 51% related to fish samples (Figure 1).

### 2.3. Tolerance to Toxic Metals

The toxic metal tolerance patterns for the isolated bacteria are presented in terms of MIC_50_, MIC_90_ and the range of MICs for the isolated bacteria previously grouped accordingly to the number of antimicrobial classes for which they showed to be resistant to: MDR (resistance to more than three classes), RES (resistance to one–two classes) and SUS (no resistance recorded). The observed mercury tolerance was higher for MDR (MIC_90_ = 256 µg/mL) if compared to RES and SUS groups (MIC_90_ = 128 µg/mL and MIC_90_ = 64 µg/mL, respectively). The same was observed for cadmium tolerance, wherein MIC_90_ for the MDR group was 1024 µg/mL, and 512 µg/mL for both the RES and SUS groups. The isolates from all three groups presented a high tolerance to copper, nickel, zinc and chromium, with an MIC_50_ of ≥1024 µg/mL (Table 4).

### 2.4. Phenotypic Expression of Efflux Mechanisms

The expression of the efflux mechanisms was determined by their ability to pump ethidium bromide out of the cell and, therefore, not emit a fluorescence when under UV lighting. Similarly, to that which was previously described for the toxic metal tolerance, the expression of the efflux mechanisms is presented in terms of the minimal concentration for the fluorescence for up to 50% of the tested samples (MCF_50_), up to 90% of the tested samples (MFC_90_) and the range of the recorded MFC. In addition, the isolated bacteria were grouped accordingly to the number of antimicrobial classes for which they showed to be resistant: MDR (resistance to more than three classes), RES (resistance to one–two classes) and SUS (no resistance recorded). Thus, the MFC_90_ was 2.5 µg/mL for the MDR group and 2 µg/mL for the RES and SUS groups. In general, although the results may indicate a similar efflux activity for all the groups of enterobacteria evaluated, MFC_50_ as well as the range in the EtBr concentration, which caused any bacterial sample to emit a fluorescence, were lower in the NS or SUS groups (Table 5).

## 3. Discussion

With the increasing consumption of ASF worldwide, meeting microbiological safety standards is important for producers, retailers and consumers alike. According to Brazilian legislation, the maximum count for mesophilic aerobic bacteria is 10^6^ CFU/g for poultry, beef and pork, which indicates that the microbial counts observed in this study meet the recommended values [18]. Even though the European Union has no similar legislation for poultry and fish, the means for mesophilic aerobes encountered here are still considerable. By the other hand, studies have reported that the lack of proper hygiene during by workers meat handling is a major source of Gram-positive cocci contamination for these products [19,20,21]. Taking that fish fillets (as those sampled) are sold pre-packaged by fishmongers and therefore would suffer less handling than poultry, pork and beef, which are kept open in refrigerated displays in butcher shops, the findings regarding Gram-positive counts in this study would corroborate the literature once these bacteria group counts were lower in fish samples.

According to the presumptive biochemical identification proposed by Procop et al., the assessment of glucose fermentation, sucrose and/or lactose fermentation, CO_2_ production during glucose fermentation and hydrogen sulfide production allow for an estimate of enterobacteria [22]. This estimate enabled an association between the relevant genera for human health and is of an environmental significance. It is possible that the proximity of the two groups in the food matrix could facilitate the transference of antimicrobial resistance molecular markers from one another. Genus such as *Lelliottia*, typically non-pathogenic bacteria found mainly in water and plants, have been known to harbor a resistance to genes which are likely of a natural origin, and these could be transferred to other species of mesophilic aerobe and facultatively anaerobic Gram-negative rods [23,24]. The observed would, thus, be implicated on the emergence of new resistant pathogenic strains.

A resistance to cefazolin was the most frequent worrisome phenotype observed, followed by ampicillin, found in 39.1% and 30.5% of the isolates, respectively. Although high, these rates of resistance are still lower compared to similar studies in India, China, Ghana and Uganda [2,25,26,27]. The use of penicillins and cephalosporins as growth promoting agents is not allowed in animal husbandry in Brazil, which indicates that the selective pressure responsible for the prevalence of this phenotype would be derived from a different source, especially considering the One Health approach. As for other classes of antimicrobial groups, the results are comparable to studies conducted in Spain, Indonesia and Nigeria [28,29,30].

Nonetheless, some of the meat and fish samples, as those evaluated in this study are a potential source for the spread of AMR microorganisms, are simultaneously resistant to up to six of the antimicrobials tested. The classification proposal for an antimicrobial resistance in MDR, extensively drug-resistant (XDR) and pan drug-resistant (PDR) by Magiorakos et al., are widely recognized and scientifically accepted. Our results indicate that 22.7% of the isolated microorganisms in the present study express a phenotype compatible to a multiple resistance to antimicrobials of a clinical relevance [17]. One isolate showed a resistance to only aztreonam and ceftazidime, and despite not fitting in the mentioned classification for MDR, it could be interpreted as being able to produce the extended spectrum beta-lactamases or AMPc and be considered MDR by the classical definition (Supplementary Table S1). It is worth noting that not all the antimicrobial categories proposed by the mentioned authors were tested, hindering the identification of XDR and PDR strains. Additionally, while a species identification was not relevant nor one of the goals in the present study, the resistance to antimicrobials found could be associated with the existence of intrinsically resistant bacterial species (e.g., *Citrobacter freundii*, *Klebsiella aerogenes* and others) [31]. This does not exempt the fact that, in spite of the possibility of being tied to an intrinsic resistance, molecular markers conferring a resistance to the antibiotics tested are still present and could be transferred through mobile genetic elements.

The antimicrobial susceptibility patterns observed for the bacteria isolated from each of the different types of meat were very similar, wherein a resistance to ampicillin, amoxicillin/clavulanic acid, cefoxitin and cefazolin was the most prevalent. Especially in the One Health perspective, the patterns found may suggest that the indiscriminate use of these drug classes, whether in human or veterinary medicine, associated with the use and disposal of xenobiotics in the environment could be related to a strong selective pressure in the different settings of animal husbandry [16]. There is a link between these settings and the aquatic ecosystem where fish farming occurs as well, with studies showing that water has an important role in connecting environments [32]. Hence, aquatic ecosystems play an important part in this scenario, allowing for an environmental network that contributes to the movement of antimicrobial resistance molecular markers and determinants. There is evidence that environmental, human and animal bacteria can harbor similar types of gene sequences and/or identical plasmid-borne resistance genes, even other mobile genome structures. These findings altogether reinforce that the environmental drug-resistant bacteria and their molecular markers of resistance are potentially transmitted to humans and their associated microbiota [33,34].

Adding to this scenario, it is also important to point out the common practice of storing unpacked pork, beef and poultry in the same refrigerated displays in Brazilian butcher shops, as well as using the same equipment to process the meats (cutting boards, knives, grinders and others). This might be, by the other hand associated with an eventual cross-contamination that may result in the similarities between the microbiota found in each type of meat. While that may be the case, it was clear that poultry was the most risk-associated type of meat, harboring the highest amounts of MDR *Enterobacteriaceae* (34.3%). This suggestion is in accordance with the findings of Skocková et al., wherein upon an analysis of *Escherichia coli* isolates derived from poultry, pork, beef and venison, it was also concluded that poultry was the most risk-associated meat in terms of the antibiotic resistance [35]. Anyway, to assess if a resistance to certain types of antimicrobials was characteristic of one or more of the meats, we investigated whether individuals could be grouped according to their susceptibility patterns with a hierarchical clustering approach. Due to high homogeneity between the three resulting groups (A1, A2 and A3), it was not possible to separate the meats considering said susceptibility patterns. This indicates that the resistance to the drugs tested in the present study is so widespread that the chain productions seem to be intrinsically related to the One Health discussions and the role of the surrounding ecosystems in the widespread antimicrobial resistance, especially soil and water.

The occurrence of metal tolerant foodborne pathogens has been reported by multiple studies. It is accepted that an antimicrobial co-resistance and cross-resistance phenomena (resistance borne by adjacent genetic markers in mobile genome such as integrons and the multiple-resistance physiological mechanisms related to one genetic marker, respectively) may be screened by a toxic metal tolerance [36,37,38]. Nonetheless, the lack of technical standards for such experimental designs hinders a data comparison. In order to bypass this limitation, the present toxic metal tolerance assays were performed according to the agar dilution technique as recommended by the CLSI and already published in previous studies by the author of this paper’s research group [31,39,40].

The isolates derived from all types of meat studied exhibited a high tolerance to the toxic metals zinc (Zn), nickel (Ni), chromium (Cr) and copper (Cu). Animal husbandry and agriculture are among the greatest sources of an environmental toxic metal contamination, which can also be carried throughout the food production chain [41]. It is possible that a high selective pressure occurs in the environment due to the mentioned metals’ build-up, which would result in the observed results. Indeed, Cu and Zn are frequently used in animal husbandry as antiseptics, feed supplements and in the prevention of piglet diarrhea [42]. The supplementation of Cr in multiple species is also common practice and has demonstrated a wide variety of positive physiological effects, such as improvements in fertility, fecundity and stress [43]. Lastly, Ni is also supplemented in animal feed and had been shown to improve the performance of growing cattle by increasing the urease activity [44].

Studies have also shown that the occurrence of MDR is positively related to a tolerance to toxic metals in microorganisms [45]. Indeed, in the present study it was found that MIC_90_ for mercury and cadmium in MDR were higher compared to those of the RES and SUS groups. As stated before, molecular markers such as those conferring tolerance to mercury are often found in transposons and integrons alongside antimicrobial-resistant genes in Gram-negative bacteria [46,47]. Moreover, a tolerance to cadmium is usually related to efflux mechanisms, which also plays an important part in the resistance by MDR microorganisms [48].

Bacterial efflux systems pose complex therapeutical challenges regarding the resistance to antibiotics due to their amount and diversity [49]. These systems have a frequently overlapping substrate recognition, and a single pump may be related to the expelling of several biocides, toxic ions and antibiotics [50]. Upon a phenotypic expression analysis of the efflux pumps in the bacteria isolates in this study, we observed a high efflux activity for all the groups tested, making the activity for the MDR group slightly higher. This could be related to the fact that the efflux pumps being expressed may not be linked only to antibiotic resistance, but rather to the extrusion of other compounds and ions. It is also worth noting that we only tested representatives of clinically relevant antibiotic classes in this study, and it is possible that the isolates present in the non-susceptible and susceptible groups could be resistant to other representatives of said classes. Further studies are needed so as to better characterize the nature of the efflux mechanisms expressed and the impact of their expression in the environment.

## 4. Materials and Methods

### 4.1. Sample Collection and Microbiological Quantification

Between 2018 and 2020, samples of beef knuckle (*n* = 21), chicken breast (*n* = 21), pork shank (*n* = 21) and fish fillet (tilapia, *n* = 21) were randomly acquired in 23 retail establishments located in Juiz de Fora (Southeast Brazil). Portions of 25 g were aseptically weighed, grounded and homogenized in 225 mL of sterile buffered peptone water for 1 min. Serial dilutions of the homogenate until 10^−4^ were performed and 0.1 mL of each dilution was spread in Petri dishes containing the following culture media: blood agar (BA) (Kasvi, Brazil) for the total viable counts of the mesophilic aerobic bacteria colony forming units (CFUs); eosin methylene blue agar (EMBA) (Kasvi, Brazil) for the selective count of the Gram-negative rods and mannitol salt agar (MSA) (Kasvi, Brazil) for the selective count of Staphylococci. All the plates were incubated at 37 °C for 24 h and counting was performed in plates containing between 30 and 300 colonies. After that, 3 to 5 representative colonies of each morphotype present in the plates were selected and cultivated in brain–heart infusion agar (BHI) (Kasvi, Brazil) for a further analysis regarding their morphotinctorial aspects using Gram staining, and then stored at −20 °C.

### 4.2. Characterization of Enterobacteria and Antimicrobial Susceptibility Patterns

Enterobacteria, which included representative members of *Enterobacteriaceae*, *Morganellaceae* and *Yersiniaceae* families, were screened among Gram-negative rods based on their ability to ferment glucose, sucrose and/or lactose, as well as gas production which included differential H_2_S [22]. The antimicrobial susceptibility testing of the isolated enterobacteria was conducted by the disc diffusion technique using Müeller–Hinton agar (Kasvi, Brazil) and antimicrobial impregnated discs (DME—Brazil) [51]. The tested antimicrobial agents were: ampicillin (AMP, 10 µg), aztreonan (ATM, 30 µg), amoxicillin/clavulanic acid (AMC, 30 µg), amikacin (AMI, 30 µg), gentamicin (GEN,10 µg), ciprofloxacin (CIP, 5 µg), levofloxacin (LEV, 5 µg), cefazolin (CFZ, 30 µg), cefoxitin (CFO, 30 µg), ceftazidime (CAZ, 30 µg), ceftriaxone (CRO, 30 µg), cefepime (CPM, 30 µg), chloramphenicol (CLO, 30 µg), meropenem (MPM, 10 µg), sulfamethoxazole/trimethoprim (SUT, 25 µg) and tetracycline (TET, 30 µg). After incubation at 37 °C for 24 h, the zones of the growth inhibition were measured, and the values compared to the 29th edition of the CLSI M100 protocol for the determining of the antimicrobial susceptibility patterns [31]. *Pseudomonas aeruginosa* ATCC 27853, *Escherichia coli* ATCC 25922, *Escherichia coli* ATCC 35218 and *Staphylococcus aureus* ATCC 25923 were used for the quality control, according to the guidelines [31]. Considering the antimicrobial resistance patterns, the isolated bacteria were clustered as multidrug-resistant (MDR) according to the criteria proposed by Magiorakos et al. (2012). Further, it was used to determine which isolates could be considered multidrug-resistant and evaluate the sample’s contaminating source level of risk [17].

In an epidemiological typing approach, to better characterize the relationships between the bacteria source and antimicrobial susceptibility patterns, a binary matrix of resistance or susceptibility to the tested drugs was drawn to enable a similarity phenogram based on a hierarchical clustering analysis by UPGMA (Unweighted Pair Group Method with Arithmetic mean) with a bootstrap of 1000×.

### 4.3. Tolerance to Toxic Metals

The minimum inhibitory concentration (MIC) for the toxic metals was determined through the agar dilution method according to the Clinical and Laboratory Standards Institute guidelines for antimicrobial drugs [31]. However, since there are no standards for the toxic metal susceptibility, the results were classified as a high or low tolerance to toxic metals.

The selected metals were chosen according to their environmental availability and biological relevance: nickel (NiCl_2_·6H_2_O) (Vetec, Brasil), zinc (ZnSO_4_·7H_2_O) (Vetec, Brasil), mercury (HgCl_2_) (Vetec, Brasil), cadmium (CdCl_2_·H_2_O) (Vetec, Brasil), chromium (Cr (NO_3_)_3_) (Vetec, Brasil) and copper (CuSO_4_) (Vetec, Brasil). Sterile solutions of the toxic metals were added to Mueller–Hinton agar plates to achieve the final concentrations ranging from 0.6 to 1024 μg/mL. These plates were inoculated using a multiple inoculator and incubated at 37 °C for 24 h away from light. The assay was performed in duplicate and plates containing pure Mueller–Hinton agar were used as the control.

### 4.4. Phenotypic Expression of Efflux Mechanisms

The expression of the efflux mechanisms was assessed through the cartwheel method with modifications [52]. Suspensions corresponding to 0.5 in the McFarland scale were prepared with each of the isolates in the study, corresponding to 1.5 × 10^8^ CFU/mL. An inoculation was carried out with a multiple inoculator in Müeller–Hinton agar plates containing varying concentrations of Ethidium Bromide (Promega, Masidon, WI, USA) (0.5 μg/mL, 1.0 μg/mL, 1.5 μg/mL, 2.0 μg/mL and 2.5 μg/mL), which were later incubated at 37 °C for 24 h away from light. Afterwards, the results were analyzed using UV light in a transillumination system. The assay was performed in duplicate and plates containing pure Müeller–Hinton agar were used as the control.

### 4.5. Statistical Analysis

During data analysis, the Dixon test was used to exclude the outliers present in each group so as to avoid an overestimation of the means. A statistical significance was calculated using the Kruskal–Wallis multiple comparisons test (*p* = 0.05) and the Steel–Dwass post hoc test was applied for a comparison of the individual means.

## 5. Conclusions

Overall, the present study offers insights on the occurrence of MDR and the antimicrobial resistance levels of mesophilic aerobe and facultatively anaerobic Gram-negative rods, presumptively characterized as enterobacteria in the most commonly consumed types of meat and fish in Brazil. Even though high levels of resistance to the antimicrobial drugs tested were not found in general, a resistance to drugs of clinical importance was still present, which raises concerns regarding the safety for the consumption of the products. Poultry proved to be the highest risk-associated type of meat among those tested, highlighting the fact that information regarding the microbiota of retail meats is of utmost importance so as to develop a sustainable One Health strategy to tackle the issue of AMR. The fact that meats can be a source of MDR microorganisms constitutes a serious public health and environmental issue.

## Figures and Tables

**Table 1:** Mean Bacterial viable counts (log10 CFU/g) in meat and beef samples considering different selective culture for Gram-positive and Gram-negative human putative bacteria.

Source		BA ^1^	EMBA ^2^	MSA ^3^
Meat	Beef	5.69	4.94	5.00
	Pork	5.82	4.95	5.28
	Chicken	6.04	4.85	4.97
Fish		6.14	5.16	3.77 *
Total (mean count; sd ^4^)		5.92; ±0.17	4.97; ±0.11	4.75; ±0.58

^1^ BA = blood agar—culture for total mesophilic facultatively anaerobic bacteria; ^2^ EMBA = eosin methylene blue agar—selective culture for Gram-negative rods; ^3^ MSA = mannitol salt agar—selective culture for Gram-positive cocci; ^4^ sd = standard deviation. * *p* < 0.05 when compared the mean values to the other samples in the same selective medium.

**Table 2:** Antimicrobial susceptibility patterns (%) of mesophilic facultatively anaerobic Gram-negative rods presumptively characterized as enterobacteria isolated from meat and fish samples.

Antimicrobial Drugs	Bacterial Source								Total (*n* = 436)	
	Beef (*n* = 91)		Pork (*n* = 112)		Chicken (*n* = 91)		Fish (*n* = 136)			
	R	I	R	I	R	I	R	I	R	I
AMP	31.9	6.5	29.5	5.4	45.0	11	20.6	5.9	30.5	6.0
ATM	0.0	0.0	0.0	0.0	3.3	0.0	2.21	0.0	1.4	0.0
AMC	22.0	3.2	17.9	5.4	26.3	6.5	10.3	2.2	18.1	3.0
AMI	1.1	1.0	0.0	0.0	0.0	0.0	0.0	0.0	0.2	0.0
GEN	0.0	0.0	0.0	0.0	0.0	0.0	0.0	0.0	0.0	0.0
CIP	1.1	0.0	3.6	1.8	2.2	0.0	0.0	0.0	1.6	0.5
LEV	1.1	0.0	1.8	0.0	2.2	0.0	0.0	0.0	1.1	0.0
CFZ	46.2	0.0	33.5	0.0	46.1	0.0	34.5	0.0	39.1	0.0
CFO	23.1	4.4	17.0	2.7	23.0	3.2	11.0	2.9	17.7	3.3
CAZ	0.0	0.0	0.0	0.0	3.3	1.0	1.5	0.7	1.2	0.2
CRO	0.0	0.0	0.0	0.0	0.0	0.0	1.5	0.0	0.5	0.0
CPM	0.0	0.0	0.0	0.0	0.0	0.0	1.5	0.0	0.5	0.0
CLO	2.2	0.0	5.4	2.7	4.4	0.0	0.7	0.0	3.3	0.5
MPM	0.0	0.0	0.0	0.0	0.0	0.0	0.0	0.0	0.0	0.0
SUT	2.2	0.0	5.4	0.0	2.2	0.0	2.2	0.0	3.0	0.0
TET	6.6	0.0	8.9	1.8	5.5	2.2	4.4	2.2	6.3	1.6

R = % of resistance; I = intermediate resistance; AMP: ampicillin; ATM: aztreonam; AMC: amoxicillin/clavulanic acid; AMI: friend; GEN: gentamicin; CIP: ciprofloxacin; LEV: levofloxacin; CFZ: cefazoline; CFO: cefoxitin; CASE: ceftazidime; CRO: ceftriaxone; CPM: cefepime; CLO: chloramphenicol; MPM: meropenem; SUT: sulfazotrim; TET: tetracycline.

**Table 3:** Distribution of simultaneous antimicrobial-resistance phenotype (different drug pharmacological classes) among mesophilic facultatively anaerobic Gram-negative rods isolated from meat and fish samples.

No. of Resistance to Drugs ^a^	% of Resistant Isolates According to Bacteria Source				Total (%)
	Beef	Pork	Chicken	Fish	
1	41.8	33.0	26.4	30.1	32.3
2	9.9	9.8	11.0	6.6	8.1
3	5.5	10.7	8.8	4.4	7.9
4	13.2	9.8	20.9	5.1	11.4
5	1.1	2.7	3.3	2.2	2.3
6	1.1	1.8	1.1	*nd* ^c^	0.9
% of MDR ^b^	20.9	25.0	34.3	11.7	22.7

^a^ Antimicrobial resistance phenotypes simultaneously observed for a single bacteria isolate; ^b^ MDR = multidrug-resistant bacteria according to classification criteria proposed by Magiorakos et al. (2012); ^c^ data not detected.

**Table 4:** Toxic metal tolerance patterns (µg/mL) of mesophilic aerobe and facultatively anaerobic Gram-negative rods presumptively characterized as enterobacteria isolated from meat and fish samples.

Metal	MDR			RES			SUS		
	MIC_50_	MIC_90_	Range	MIC_50_	MIC_90_	Range	MIC_50_	MIC_90_	Range
Ni	1024	>1024	1024–>1024	>1024	>1024	512–>1024	1024	>1024	256–>1024
Zn	>1024	>1024	512–>1024	>1024	>1024	128–>1024	1024	>1024	256–>1024
Hg	32	256	2–256	16	128	2–256	32	64	8–256
Cd	512	1024	128–1024	256	512	128–512	256	512	32–1024
Cr	>1024	>1024	>1024	>1024	>1024	>1024	>1024	>1024	>1024
Cu	>1024	>1024	>1024	>1024	>1024	>1024	1024	1024	256–1024

Ni = nickel (NiCl_2_·6H_2_O); Zn = zinc (ZnSO_4_·7H_2_O); Hg = mercury (HgCl_2_); Cd = cadmium (CdCl_2_·H_2_O); Cr = chromium (Cr(NO_3_)_3_); Cu = copper (CuSO_4_); MDR = bacteria group for which resistance was observed to more than 3 antimicrobial classes; RES = bacteria group for which resistance was observed to 1–2 antimicrobial classes; SUS = bacteria group for which antimicrobial resistance was not recorded.

**Table 5:** Expression of efflux mechanisms (µg/mL) in terms of bacterial ability to pump ethidium bromide (EtBr) out of the cell and do not emit fluorescence when under UV lighting among mesophilic aerobe and facultatively anaerobic Gram-negative rods presumptively characterized as enterobacteria isolated from meat and fish samples.

EtBr Efflux Measure ^a^	MDR	RES	SUS
MFC_50_	2.0	1.5	1.5
MFC_90_	2.5	2.0	2.0
MFC Range	1.0–2.5	1.0–2.5	0.5–2.5

^a^ MFC = minimal fluorescence concentration, up to 50% of the tested samples (MCF_50_), up to 90% of the tested samples (MFC_90_) and range of recorded MFC. MDR = bacteria group for which resistance was observed to more than 3 antimicrobial classes; RES = bacteria group for which resistance was observed to 1–2 antimicrobial classes; SUS = bacteria group for which antimicrobial resistance was not recorded.

## Data Availability

Not applicable.

## Supplementary Materials

The following are available online at https://www.mdpi.com/article/10.3390/antibiotics11121677/s1, Table S1: Antimicrobial resistance phenotypes observed among mesophilic facultatively anaerobic Gram-negative rods presumptively characterized as enterobacteria isolated from meat and fish samples in Southern Brazilian retail markets.
