# Supplementary material for: Physiological Characteristics of Putative Enterobacteria Associated with Meat and Fish Available in Southern Brazilian Retail Markets: Antimicrobial Susceptibility, Toxic Metal Tolerance and Expression of Efflux Pumps
Source: Antibiotics (Basel). 2022 Nov 22;11(12):1677. doi: 10.3390/antibiotics11121677 (PMC9774923; doi:10.3390/antibiotics11121677)
Supplement: Supplementary file 1 [file antibiotics-11-01677-s001.zip › antibiotics-1994125-supplementary.pdf]

antibiotics

MDPI

Article

Physiological Characteristics of Putative Enterobacteria Associated with Meat and Fish Available in Southern Brazilian Retail Markets: Antimicrobial Susceptibility, Toxic Metal Tolerance and Expression of Efflux Pumps

Renata da Costa Barros Silva, Jéssica Andrade, Vanessa Cordeiro Dias, Jéssica Carla Sequeto, Nayara Felga Santos, Vânia Lúcia da Silva and Cláudio Galuppo Diniz *

Laboratory of Microbial Physiology and Molecular Genetics, Center for Studies in Microbiology, Department of Parasitology, Microbiology and Immunology, Federal University of Juiz de Fora, Juiz de Fora 36036-330, Brazil
* Correspondence: claudio.diniz@ufjf.br

Supplementary Information

Table S1 Antimicrobial resistance phenotypes observed among mesophilic facultatively anaerobic Gram-negative rods presumptively characterized as enterobacteria isolated from meat and fish samples in Southern Brazilian retail markets.

AMR phenotype	Number of bacteria isolates (n) and frequency (%)				
	Chicken (n= 91)	Beef (n= 91)	Pork (n= 112)	Fish (n= 136)	Total (n= 436)
CFZ	12 (13.2)	25 (27.5)	19 (17.0	30 (22.1)	86 (20.0)
AMP	11 (12.1)	8 (8.8)	9 (8.0)	7 (5.1)	34 (7.9)
CFO	-	4 (4.4)	4 (3.6)	1 (0.7)	9 (2.0)
TET	-	1 (1.1)	3 (2.7)	1 (0.7)	5 (1.1)
SUT	-	-	2 (1.8)	1 (0.7)	3 (0.7)

ATM	1 (1.1)	-	-	1 (0.7)	2 (0.4)
AMP-CFZ	4 (4.4)	1 (1.1)	1 (0.9)	5 (3.7)	11 (2.5)
CFZ-CFO	3 (3.3)	2 (2.2)	2 (1.8)	1 (0.7)	8 (1.8)
AMP-TET	-	1 (1.1)	2 (1.8)	1 (0.7)	4 (0.9)
AMC-AMP	1 (1.1)	1 (1.1)	2 (1.8)	-	3 (0.7)
AMP-SUT	1 (1.1)	-	1 (.09)	-	2 (0.4)
SUT-TET	-	1 (1.1)	1 (0.9)	-	2 (0.4)
CFO-SUT	1 (1.1)	-	-	-	1 (0.2)
AMI-CLO	-	1 (1.1)	-	-	1 (0.2)
AMP-CFO	-	1 (1.1)	-	-	1 (0.2)
CFO-TET	-	-	1 (0.9)	-	1 (0.2)
ATM-CAZ	1 (1.1)	-	-	-	1 (0.2)
AMC-CFZ-AMP	4 (4.4)	4 (4.4)	9 (8.1)	4 (2.9)	21 (4.8)
AMC-CFO-AMP	-	-	-	-	5 (1.1)
AMP-AMC-CFZ	-	-	-	-	7 (1.6)
AMP-CLO-SUT	1 (1.1)	-	1 (0.9)	-	2 (0.4)
AMP-AMC-CFO	1 (1.1)	-	-	-	1 (0.2)
AMP-CFZ-CFO	1 (1.1)	-	-	-	1 (0.2)
CFZ-SUT-TET	-	1 (1.1)	-	-	1 (0.2)
AMP-CFZ-TET	-	-	1 (0.9)	-	1 (0.2)
AMP-CIP-CLO	-	-	1 (0.9)	-	1 (0.2)
AMP-SUT-TET	-	-	-	1 (0.7)	1 (0.2)

CLO-SUT-TET	-	-	-	1 (0.7)	1 (0.2)
AMP-AMC-CFZ-CFO	14 (15.4)	12 (13.2)	10 (8.9)	6 (4.4)	42 (9.7)
AMP-CFZ-CFO-CLO	2 (2.2)	-	1 (0.9)	-	3 (0.7)
AMP-AMC-CFZ-TET	1 (1.1)	-	-	-	1 (0.2)
AMP-CIP-LEV-TET	1 (1.1)	-	-	-	1 (0.2)
AMP-AMC-CFZ-CAZ	1 (1.1)	-	-	-	1 (0.2)
AMC-CFZ-CFO-TET	-	-	-	1 (0.7)	1 (0.2)
AMP-AMC-CFZ-CFO-TET	1 (1.1)	1 (1.1)	1 (0.9)	1 (0.7)	4 (0.9)
AMP-AMC-CFZ-CLO-SUT	-	-	2 (1.8)	-	2 (0.4)
ATM-CPM-CFO-CAZ-CRO	-	-	-	2 (1.5)	2 (0.4)
AMP-AMC-ATM-CFZ-CAZ	1 (1.1)	-	-	-	1 (0.2)
AMP-AMC-CFZ-CFO-CLO	1 (1.1)	-	-	-	1 (0.2)
AMP-AMC-CIP-CLO-LEV-TET	1 (1.1)	1 (1.1)	2 (1.8)	-	4 (0.9)

AMP = ampicillin, 10 μg); ATM = aztreonan, 30 μg; AMC = amoxicillin/clavulanic acid, 30 μg, AMI = amikacin, 30 μg, GEN gentamicina, 10 μg; CIP = ciprofloxacin, 5 μg; LEV = levofloxacin, 5 μg; CFZ = cefazolin, 30 μg; CFO = cefoxitin, 30 μg; CAZ = ceftazidime, 30 μg; CRO = ceftriaxone, 30 μg; COM = cefepime, 30 μg; CLO = chloramphenicol, 30 μg; MPM = meropenem, 10 μg; SUT = sulfamethoxazole/trimethoprim, 25 μg; TET = tetracycline, 30 μg.
